# Projected Loss of a Salamander Diversity Hotspot as a Consequence of Projected Global Climate Change

**DOI:** 10.1371/journal.pone.0012189

**Published:** 2010-08-16

**Authors:** Joseph R. Milanovich, William E. Peterman, Nathan P. Nibbelink, John C. Maerz

**Affiliations:** 1 D.B. Warnell School of Forestry and Natural Resources, University of Georgia, Athens, Georgia, United States of America; 2 Department of Biological Sciences, University of Missouri, Columbia, Missouri, United States of America; Duke University, United States of America

## Abstract

**Background:**

Significant shifts in climate are considered a threat to plants and animals with significant physiological limitations and limited dispersal abilities. The southern Appalachian Mountains are a global hotspot for plethodontid salamander diversity. Plethodontids are lungless ectotherms, so their ecology is strongly governed by temperature and precipitation. Many plethodontid species in southern Appalachia exist in high elevation habitats that may be at or near their thermal maxima, and may also have limited dispersal abilities across warmer valley bottoms.

**Methodology/Principal Findings:**

We used a maximum-entropy approach (program Maxent) to model the suitable climatic habitat of 41 plethodontid salamander species inhabiting the Appalachian Highlands region (33 individual species and eight species included within two species complexes). We evaluated the relative change in suitable climatic habitat for these species in the Appalachian Highlands from the current climate to the years 2020, 2050, and 2080, using both the HADCM3 and the CGCM3 models, each under low and high CO_2_ scenarios, and using two-model thresholds levels (relative suitability thresholds for determining suitable/unsuitable range), for a total of 8 scenarios per species.

**Conclusion/Significance:**

While models differed slightly, every scenario projected significant declines in suitable habitat within the Appalachian Highlands as early as 2020. Species with more southern ranges and with smaller ranges had larger projected habitat loss. Despite significant differences in projected precipitation changes to the region, projections did not differ significantly between global circulation models. CO_2_ emissions scenario and model threshold had small effects on projected habitat loss by 2020, but did not affect longer-term projections. Results of this study indicate that choice of model threshold and CO_2_ emissions scenario affect short-term projected shifts in climatic distributions of species; however, these factors and choice of global circulation model have relatively small affects on what is significant projected loss of habitat for many salamander species that currently occupy the Appalachian Highlands.

## Introduction

Understanding how species distributions and patterns of diversity shift with changing climates has been a long-standing theme of ecology that has grown less academic with the specter of rapid climate change. Not surprisingly, there is an increasing effort to project the effects of climate change on species' distributions and regions of high biodiversity [1], [2], [3], [4], [5]. Knowing whether particular species or hotspots of biodiversity are vulnerable to decline is important to planning management actions and understanding how ecosystem functions may change [6].

Species distribution modeling is one tool for evaluating the potential impact of climate change on the distributions of biota [7], [8]. Distribution models characterize dimensions, generally mean climatic variables, of the current realized niche of a species based on presence-absence data and then use future climate forecasts to project changes in the distribution of suitable habitat for a species. Climate-driven species distribution models have several limitations including exclusion of other biotic, physiological, and geographic controls on a species' distribution. Additionally, these models cannot mechanistically account for the role of climate in determining species distributions or quantify the limits of species abilities to migrate. Furthermore, this technique ignores the capability of evolutionary change to compensate for species responses to changing climate and they assume reliance upon credible climatic projections by assuming that the “suitable” habitat is saturated and the data input into models is accurate [9], [10], [11], [12], [13], [14]. Projections from climate distribution modeling are also dependent upon the global circulation model selected, how well that model can be downscaled to predict local climate [15], and assumptions about future atmospheric CO_2_ levels. To deal with the potential limitations of model projections, increasingly studies often take an ensemble forecasting approach by modeling a number of future scenarios that bracket ranges of model assumptions or predicted climate change scenarios [16]. The most common approach is to integrate different global circulation models and CO_2_ emissions scenarios and forecast out to multiple future time points.

A potential criticism of forecasts from species distribution modeling is the self-fulfilling nature of the endeavor. Based on relationships between climate variables at sites occupied by a species, climate distribution models such as Maxent [17] subsequently provide a continuous probability surface which can be classified (based on a threshold) into suitable or non-suitable climatic space. The user determines the threshold, which is often set to a single value, and then generates a current climate-driven distribution to best fit the known species distribution [18]. In other words, the user makes the species' distribution a strict function of the variables that are put into the model (e.g., climate, land cover, soil type). Because the threshold may be a somewhat arbitrary cutoff depicting presence/absence of a species, applying a more liberal threshold in climate distribution models may dampen projected effects of climate change on species' distributions, such as the inability to cross geographic barriers.

We used a combination of Global Circulation Models (GCM), atmospheric CO_2_ scenarios, and both strict and liberal model thresholds to generate a range of projected shifts in potential suitable climatic habitat for plethodontid salamanders in the southern Appalachian region of the eastern United States. Areas with high biodiversity or endemism are of high conservation value, and the Appalachian Highlands are regarded as a biodiversity hotspot with some of the most biologically diverse forests and freshwater systems in the United States [19]. At broad spatial scales, amphibian diversity is related strongly to the direct and indirect (via net primary production) effects of climate and regional phylogeography [20], [21]. The Appalachian Highlands are a global hotspot for salamander diversity, nearly all of which is determined by the family Plethodontidae [22]. Plethodontid distributions are determined by a number of factors including land forms (e.g., major river boundaries), history and biotic interactions such as interspecific competition [23], [24]; however, because plethodontids are lungless ectotherms, their activity, life-history traits, and consequently geographic distributions and patterns of diversity appear predominantly controlled by climate. [25], [26], [27], [28], [29]. Consistent with global patterns of amphibian diversity [20], [21], plethodontid species richness throughout the southern Appalachian Highlands is positively linked to the cool, moist montane climate [28] with most species occupying mid or high elevation climatic zones that were colonized millions of years earlier when those climatic zones occurred in valley bottoms [28], [29]. Recent evidence suggests temperature is a direct limiting factor of dispersal and range size of some species within the family [26], further supporting the use of climate-based models to examine species distributions within this family. Because plethodontid salamanders are the most abundant vertebrate predators in eastern North American forests and headwater streams and are influential in a number of ecosystem processes [30], [31], [32], [33], understanding shifts in their distributions or abundance will be important to predicting changes to ecosystem processes.

## Methods

### Species Distribution Modeling using Maximum Entropy

We developed distribution models using Maxent version 3.30a [17], [34] for 41 plethodontid species (33 individual species and eight species included within two species complexes) with distributions in the eastern United States that included a portion of the species range within the Appalachian Mountain region (defined by a geographic boundary that includes all ecoregions found within the Appalachian Highland region). The two species complexes were the *Plethodon glutinosus* complex, which was composed of six species (*P. glutinosus, P. cylindraceus, P. kentucki, P. teyahalee, P. chlorobryonis,* and *P. chattahoochee*) and the *Desmognathus fuscus* complex, composed of two species (*D. fuscus* and *D. conanti*). We treated these groups as complexes because their members were historically identified as one species but were later broken up into parapatric, morphologically cryptic species based on patterns of genetic divergence suggesting that geographic features and isolation promoted speciation [23], [35], [36], and they are nearly indistinguishable in hand (although evidence suggest there are differences in body size [37]). There are no data indicating that they function differently with regard to ecological factors such as climate. The 33 species (and complexes) represent ∼90% of plethodontid species in the southern Appalachian Highlands and ∼50% of plethodontid species occurring in the southeastern United States.

Maxent is a machine learning method that utilizes the principle of maximum entropy to model species distributions using presence-only data coupled with environmental data [34]. This approach finds a probability distribution of maximum entropy using a set of environmental variables to estimate a species' ecological niche using the defined Maxent probability distribution. For each species or species complex, current species distribution models were created using point data from two natural history databases intersected with georeferenced climatic variables. Salamander presence data were obtained from HerpNET (www.herpnet.org) and Global Biodiversity Information Facility (GBIF; www.gbif.org). To maximize model quality, only species with greater than 30 point locations were used [38]. We downloaded 1-km resolution temperature and precipitation bioclimatic layers, which are based on the 30-year period from 1960–1990, from the WorldClim database [39]. We used the 11 bioclimatic layers utilized by Rissler and Apodaca [40] in their bioclimatic distribution modeling of *Aneides flavipunctatus*, a plethodontid species distributed in the western United States. Those 11 bioclimatic layers were winnowed from a larger set of 19 variables using correlations to estimate redundancy between variables and retaining the more biologically meaningful and interpretable variables (e.g., annual mean temperature, mean temperature of the wettest quarter, and precipitation of the wettest quarter). Maxent was run from the command line using the default settings with the exception of background points. A total of 4215 target-group background data points representing localities of plethodontid salamanders in the eastern United States were used to develop an initial climatic envelope that represents the range of environmental conditions within the modeled region. In turn, this method is expected to reduce the bias inherent in our sample of museum locality data [41]. This approach uses background data (also known as pseudo-absences), chosen with the same bias as the occurrence data used, to develop the models. By using this approach we can produce an unbiased estimate of the geographic distribution of species, since the background data provides an equable sample of the environmental conditions within the region modeled.

We used a threshold approach to designate a location as climatically suitable for a species. When modeling a single species, each location modeled is represented by a probability that the location is climatically suitable for that species; however, it is logistically unfeasible to present each location as a probability of occupancy for every species modeled. Therefore, it was necessary to delineate a threshold at which a location was deemed climatically suitable or un-suitable. As was discussed in the introduction, the use of a single threshold will create a strict relationship between climate and a species' distribution, and thus potentially exaggerating the effect of climate shifts on the species' future distribution. To address this issue, we converted the continuous suitability surface [0–1 from Maxent to presence/absence (1/0)] using two model output thresholds applied by Maxent; one ‘strict threshold’ that produced a climatic distribution that closely resembled, and at times underrepresented, the species current realized distribution (fixed cumulative value 10) and one ‘liberal threshold’ that predicts a broader climatic distribution than the current realized distribution (minimum training presence). We believe that this two-threshold approach is preferable to using a single threshold because it makes our results comparable to other studies that provide predictions based on strict climatic distributions of species—thresholds that maximize the agreement between observed and predicted distributions [42], and also allows us to present model predictions that relax the assumption of strict climatic control on species' distributions.

We used null models to test the significance of each species climatic distribution model [43]. We generated 1000 sets of sample points, which were randomly drawn from the pool of 4215 background points without replacement. Since the number of presence localities varied for each species, we generated null data sets with the number of random points per distribution equal to 50, 205, 405, or 695 data points, which represent the range of presence points available to model each species. Maxent was used to calculate the area under the curve (AUC) for the 1000 null data sets to create an AUC frequency distribution. The calculated AUC for each species model was then compared to the 95 percentile AUC value of the null frequency distribution created from the representative number of sample points (50, 205, 405, or 695). A species model performs better than random and is considered significant if the calculated AUC is greater than the corresponding 95 percentile AUC of the null-distribution [43].

### Projecting Future Species Climate Distributions

Climate projections were downloaded from the WorldClim database (www.worldclim.org). Projections were derived from the IPPC 3rd Assessment [44] and were calibrated and statistically downscaled using WorldClim Version 1.4 data for current projections. The 11 bioclimatic variables were calculated using the freely available ESRI ArcInfo AML program (available at http://www.worldclim.org/bioclim.htm). We used projections for years 2020, 2050, and 2080 derived from two widely used global circulation models (GCM), the Canadian Centre for Climate Modeling and Analysis Coupled Global Climate Model (CGCM3; [45]) and the Hadley Centre for Climate Prediction and Research (HADCM3; [46]). For each GCM, we used projections of climate parameters derived from two CO_2_ emissions scenarios, A2a (medium to high emissions) and B2a (low to medium emissions) that corresponded to the IPCC Special Report on Emissions Scenarios [47]. Therefore, we developed eight spatially explicit climate model scenarios, and used the Maxent climate distribution model developed earlier to project the future climate distribution for each species to 2020, 2050 and 2080.

### Quantifying Projected Changes in Species Distributions and Richness

We compared current strict and liberal climate distribution models for each species with known distributions derived from county-level distribution maps to estimate the effect of threshold on over- or under-prediction of current known species distributions [48]. We calculated the percent overlap between modeled and county level distributions using ArcMap version 9.3 (ESRI, Redlands, CA). To measure the change in species distributions under future climate scenarios, we calculated the percentage of predicted area lost between the current and future predicted climate distribution model and compared them using the same Maxent threshold. In order to avoid the common criticism of assuming no potential for dispersal or unlimited dispersal, and to account for disjunct areas of predicted climatic habitat to which species will be unable to disperse to, we clipped all Maxent model predictions for all scenarios by the known county-level distribution buffered by 10 km. The buffer provides opportunities for future expansion by dispersal; however, we note that this is not a mechanistic adjustment and does not account for species-specific dispersal capabilities [27]. We know little concerning dispersal capabilities of plethodontid salamanders. Evidence from northern populations of the red-backed salamander (*P. cinereus*) suggests expansion at a rate of only 80 m per year [49], and other recent evidence suggests that some plethodontids may already be dispersal limited by temperature, so it is likely that any warming will further limit dispersal capabilities [26], [27]. Our 10 km buffer likely offers a liberal boundary for future migration. Each climatic and species map was projected in the World Geodetic Coordinate System of 1984 (WGS84) with a cell size of 0.0083 decimal degrees.

We also examined how well species distribution models predicted known patterns of species richness in the Appalachian Highlands region and whether different climate change model scenarios predict different effects on plethodontid diversity. To estimate patterns of species richness, we made two species richness maps based on the accumulated modeled distributions of each species or species complex using strict or liberal Maxent thresholds. Next we compared the richness of the two accumulated climate distribution models to known richness from county-based distribution maps by comparing richness values from the different distributions at 250 randomly selected points. We created the same accumulated richness maps for each of the CO_2_ X GCM model X threshold scenarios for 2020, 2050 and 2080 to examine how projected changes in individual species distributions might affect patterns of diversity within the Appalachian Highlands region.

To examine the affect of GCM, CO_2_ scenario, threshold, current range size, and distribution (latitude) on projected changes in suitable climatic habitat of species', we used a general linear model with percent habitat loss between the current suitable climatic distribution and predicted suitable climatic distributions (square root transformed) as dependent variables and GCM (Hadley or Canadian), CO_2_ emissions scenario (low or high), threshold (strict or liberal) as categorical variables, and the size of the current species range and the latitude of the distribution centroid as continuous variables. To reduce over-parameterization of the model and simplify interpretation, we restricted our analysis to main effects and two-way and three-way interaction terms. We conducted a separate analysis for each projected year (2020, 2050 and 2080). We used paired *t*-tests to compare known county-based species richness values and predicted richness values (produced by summing the richness using both the strict and liberal thresholds). Statistical analyses were conducted in STATISTICA 8.0 (Statsoft, Inc., Tulsa, OK).

## Results

The mean AUC for plethodontid distribution models based on current climate was 0.911 (range  = 0.664–0.995; median  = 0.940; Table S1), with each species' model AUC being significantly better than random (i.e., model AUC values exceeded the 95 percentile of the null AUC distributions). Model predictions more closely matched current species distributions when the liberal threshold was used (81.04%, sd = 21.25 vs. 62.83% sd = 25.89, for the strict threshold; Table S1).

**Figure 1:**
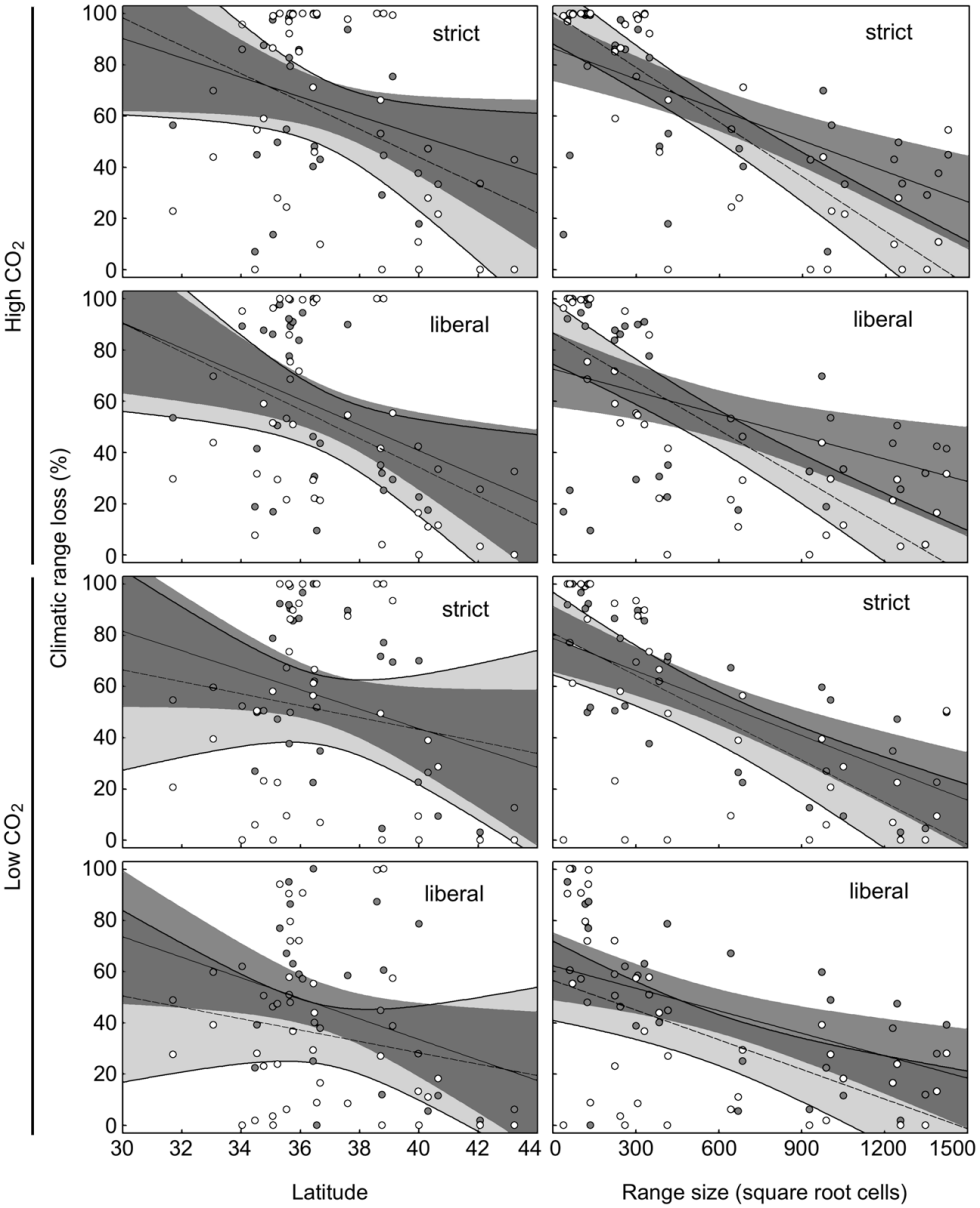
Projected percent suitable climatic habitat loss by 2020 relative to climatic and model variables. Effects of latitude and current range size on projected percent climatic habitat loss by 2020 of 35 plethodontid species/species complexes currently found within the Appalachian Highlands. Presented are percent of suitable climatic habitat losses relative to current climate distribution model for two Maxent thresholds (‘strict’ vs. ‘liberal’), two projected CO_2_ levels (‘high’ vs. ‘low’), and two global circulation models (Canadian, CGCM3  =  solid regression line with dark grey 95% confidence bands and solid points; Hadley, HADCM3  =  dashed regression line with light grey 95% confidence bands and hollow points). Darkest shading indicates regions of overlap between CGCM3 and HADCM3 confidence bands.

**Figure 2:**
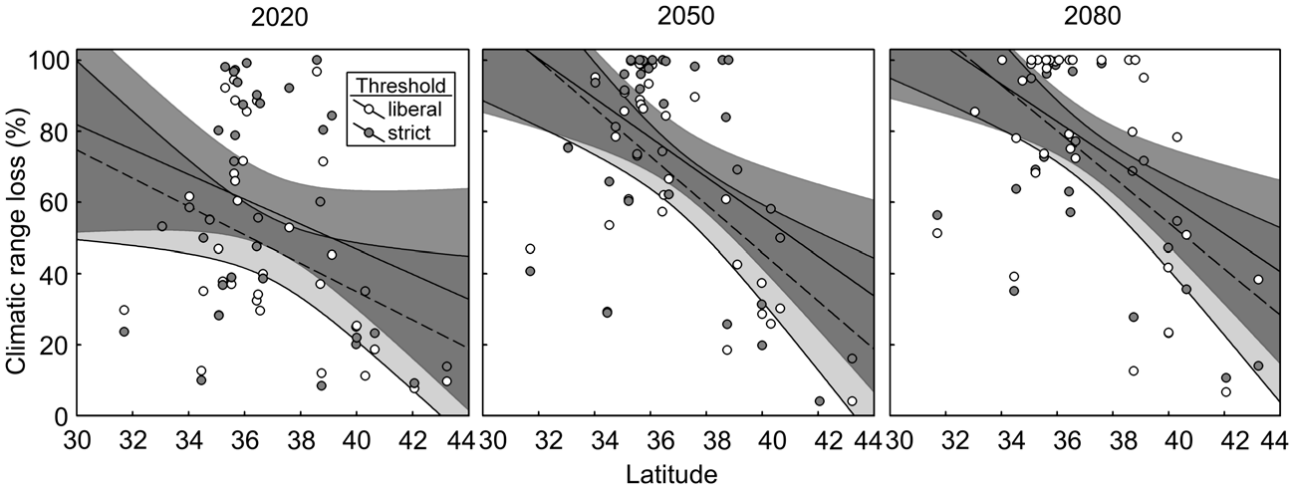
Projected percent suitable climatic habitat loss by 2020, 2050, and 2080 relative to Maxent threshold and latitude. Effect of latitude and Maxent threshold on projected percent climatic habitat loss by 2020, 2050 and 2080 for 35 plethodontid species/species complexes currently found within the Appalachian Highlands. Presented are percent suitable climatic habitat losses relative to current climate distribution model for two Maxent thresholds (‘strict’  =  solid points with a solid regression line and dark grey 95% confidence bands; ‘liberal’  =  hollow points with a dashed regression line and light grey 95% confidence bands). Darkest shading indicates regions of overlap between ‘strict’ and ‘liberal’ confidence bands.

While projected mean change in salamander suitable climatic habitat size by 2020 varied depending on threshold, assumed CO_2_ level, and current range size and latitude, even the most ‘optimistic’ model (low threshold, low CO_2_, HADCM3) projected at least a 20% reduction in suitable climatic range for more southerly distributed plethodontid species (Fig. 1; Tables S2 and S3). There were significant interactions between threshold, assumed CO_2_, and current range size and between threshold, assumed CO_2_, and centroid latitude (Table 1). Percent of suitable climatic habitat loss was highest for species with small, southerly geographic ranges under models assuming high CO_2_, and strict Maxent threshold (Fig. 1; Tables S1, S2 and S3). The effects of assumed CO_2_ and threshold were small relative to the effects of range size and latitude (Fig. 1). For later projections (2050 and 2080), only threshold and latitude significantly affected mean percent climatic habitat loss (Table 1; Fig. 2). For all time points, the percent climatic habitat loss was greatest among more southerly distributed species (range centroid 32–34° north latitude), and slightly greater for models that assume a strict Maxent threshold. The projected percent climatic habitat loss among the most southerly-distributed (range centroid 32–34° north latitude) species increased from 50–100% by 2020 to 80–100% by 2050 and 85–100% by 2080 (Fig. 2; Tables S1, S2 and S3). For mid-latitude species (range centroid 36–38° north latitude) projected percent climatic habitat loss was 40–70% by 2020 and 70–85% by 2080, and for more northerly-distributed species (range centroid 42–44° north latitude), projected percent climatic habitat loss was 0–70% (mean 20–38%) by 2020 and 0–70% (mean 30–40%) by 2080 (Fig. 2; Tables S1, S2 and S3).

**Table 1:** General linear model results.

		2020			2050			2080		
Source	df	MS	F	*P*	MS	F	*P*	MS	F	*P*
Model	1	1.037	0.213	0.645	0.025	0.012	0.914	1.835	1.009	0.316
CO_2_	1	80.185	16.467	<0.001	1.505	0.703	0.402	1.119	0.616	0.433
Threshold	1	6.227	1.278	0.259	5.001	2.341	0.127	5.699	3.136	0.078
Latitude	1	0.132	0.0271	0.869	26.626	12.443	<0.001	14.220	7.825	<0.01
Range size	1	19.884	4.085	0.044	2.580	1.206	0.273	5.454	3.001	0.084
Model*CO_2_	1	11.932	2.450	0.119	1.125	0.526	0.469	0.081	0.045	0.833
Model*Threshold	1	0.753	0.155	0.694	0.682	0.319	0.573	0.001	0.001	0.982
CO_2_*Threshold	1	0.260	0.053	0.817	1.390	0.650	0.421	0.367	0.202	0.653
Model*Latitude	1	1.194	0.245	0.621	0.013	0.006	0.937	1.934	1.064	0.303
CO_2_*Latitude	1	74.663	15.333	<0.001	1.449	0.677	0.411	1.141	0.628	0.429
Threshold*Latitude	1	7.815	1.605	0.206	5.555	2.596	0.108	6.043	3.326	0.069
Model*Range size	1	1.622	0.333	0.564	0.002	0.001	0.974	1.975	1.087	0.298
CO_2_* Range size	1	77.874	15.992	<0.0001	1.466	0.685	0.409	0.006	0.003	0.954
Threshold*Range size	1	4.389	0.901	0.343	1.681	0.785	0.376	2.835	1.560	0.212
Latitude*Range size	1	34.248	7.033	0.009	8.596	4.017	0.046	12.354	6.800	0.010
Model*CO_2_*Threshold	1	1.285	0.264	0.608	0.257	0.120	0.729	0.015	0.008	0.927
Model*CO_2_*Latitude	1	9.366	1.923	0.167	1.015	0.474	0.491	0.138	0.076	0.783
Model*Threshold*Latitude	1	0.636	0.131	0.718	0.645	0.301	0.584	<0.001	<0.001	0.987
CO_2_*Threshold*Latitude	1	0.122	0.025	0.874	1.372	0.641	0.424	<0.001	0.246	0.621
Model*CO_2_*Range size	1	9.888	2.031	0.155	0.020	0.009	0.923	0.605	0.333	0.564
Model*Threshold*Range size	1	0.044	0.009	0.925	1.085	0.507	0.477	<0.001	<0.001	1.000
CO_2_*Threshold*Range size	1	0.117	0.024	0.877	0.297	0.139	0.709	0.0315	0.017	0.895
Model*Latitude*Range size	1	3.100	0.636	0.426	0.022	0.010	0.920	1.858	1.022	0.313
CO_2_*Latitude*Range size	1	76.248	15.658	<0.0001	1.165	0.544	0.461	0.186	0.103	0.749
Threshold*Latitude*Range size	1	5.447	1.119	0.291	1.856	0.867	0.353	2.989	1.645	0.201
Error	254	4.869	–	–	2.140	–	–	1.817	–	–

Results from a general linear model investigating the factors that influenced the percent of suitable climatic habitat lost in 2020, 2050, and 2080. Data were square root transformed.

**Figure 3:**
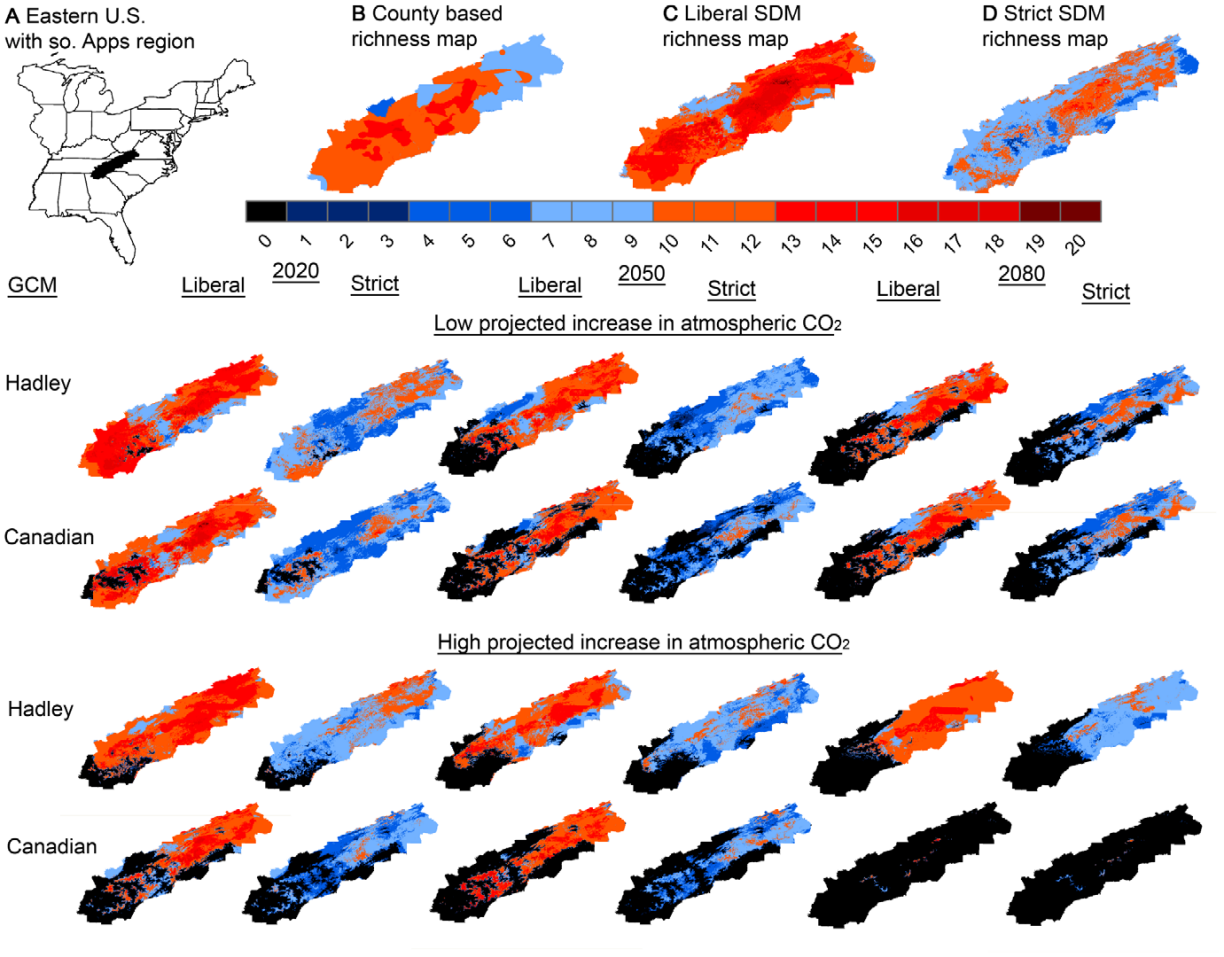
Projected species richness in the Appalachian Highlands for 2020, 2050, and 2080 under 24 climatic scenarios. Predicted species richness of southern Appalachian plethodontids under 24 scenarios by year, global circulation model, CO_2_ emissions scenario and model threshold. A) Shapefile used to create the species richness maps for the southern Appalachians. B) Species richness from county-level shapefiles. C) Current predicted suitable climatic habitat for the liberal model threshold. D) Current predicted suitable climatic habitat for the strict model threshold.

Richness estimates based on accumulated climate distribution models produced with strict and liberal thresholds differed from each other (*t* = 20.458, P<0.001; Fig. 3) and from current known richness values based on county-level distribution records. The liberal threshold over-predicted known richness for the study region (*t* = −10.106, P<0.001, mean county-level  = 10.54, mean liberal threshold  = 12.47), while the strict distribution models significantly under-predicted richness (*t* = 10.968, P<0.001, mean county-level  = 10.54, mean strict threshold  = 8.68).

Reflecting the results for species-specific projected climatic habitat losses, even the most ‘optimistic’ projections predict declines in plethodontid richness within the southern portion of Appalachian Highlands as early as 2020 (Fig. 3). 2020 richness projections for the low threshold, low CO_2_, HADCM3 model are relatively similar to current richness patterns in the region, with losses predicted only on the south-eastern fringe of the Appalachian Highlands region (Fig. 3); however, all other scenarios predict a significant loss of species in the southern highlands including the loss of all current species in the region by 2020. Over time, all model scenarios predict significant declines in species richness across the southern portion of the Appalachian Highlands with the loss of all current species from some areas under all model scenarios. Models using the more liberal Maxent threshold project the retention of high salamander richness in the central and northern portions of the Appalachian Highlands through 2080 regardless of CO_2_ level or GCM. Only the most ‘pessimistic’ models (Canadian GCM3, high CO_2_) predicting the greatest amount of warming and reduced precipitation, project a near complete loss of current species from the entire Appalachian Highland region by 2080 (Fig. 3).

## Discussion

Our modeling approach shows that, depending on model assumptions, every plethodontid salamander species currently found within the Appalachian Highlands could experience restricted climatic habitat with climate change. We note that our models do not predict the extinction of the majority of species. Rather, those species with small, southerly ranges are predicted to experience the largest declines in range size including possible extinction. Sixty percent of the species (or complexes) we modeled, which in total comprised approximately 85–90% of plethodontid species richness found within the Appalachian Highlands, have a current range smaller than 115,500 km^2^, and our models project the largest declines among those species with small ranges in the southern portion of the region. This scenario likely applies to the handful of range-limited endemics we could not analyze because of insufficient data on distributions. Projected climatic habitat declines are much smaller for species in the central and northern Appalachian Highlands region, and projected species richness remains high in the central and northern regions under a range of model scenarios. These predictions are consistent with a number of studies predicting more significant range contractions or northward shifts as a consequence to global climate change [50].

The robustness of our model predictions depends in part on the relative importance of climate versus geographic and biotic limitations on species distributions. Geographic and biotic limits on species distributions may conceal broader climatic tolerances than are reflected by a species' current distribution. Further, if biotic interactions are important in determining species distributions, and those interactions are altered by climate (e.g.[51]), then it may be difficult to predict how a species distribution may respond to climate change. The boundaries of some plethodontid species do clearly coincide with major land formations, such as rivers, or the occurrence of interspecific competitors [23], [24]. We are not aware of any data to indicate whether those species can occupy climates not represented by their current distribution. There is also evidence that interspecific competition shapes salamander phenotypes (morphology and behavior), but there is limited evidence that competition is a significant determinant of species distributions (reviewed by [29]). Evidence suggests that some high elevation species, which are strongly climatically restricted, may limit the upslope distribution of lower elevation species, but there is no evidence of the reverse. This would imply that competitive effects on species distributions are biased toward underestimating the cold tolerance of low elevation species, but not the warm tolerance of high elevation species. Therefore, interspecific competition would not confound the use of climatic models to predict range loss from climate warming.

The most compelling evidence is that, with the potential exception of some low elevation species discussed previously, most plethodontids are restricted to their current realized climatic zones. It is true that the species we modeled have persisted through several historic periods of warming, and that historic warming events were associated with periods of plethodontid diversification [52]; however, this should not be confused as evidence that historic warming events were not associated with species range contractions and extinctions. Kozak and Wiens [29] provide phylogeographic evidence that many extant plethodontid species that currently exist at mid and high-elevation climatic zones are descended from species that colonized those cool climates when they occurred at lower elevations. They suggest that species have been “strictly confined” to specific climatic zones for millions of years, and have migrated with shifting climatic zones during historic changes in climate [29]. These results are significant for several reasons. First, they suggest that it is unlikely that many plethodontid species have persisted in the same geographic location while that location has undergone significant climatic change. Rather, species migrate with their associated climatic zone during periods of climate change. Second, species currently distributed at mid and high elevations are most vulnerable to climate warming if their current climatic zone is lost because those species have limited ability to disperse through warmer valley bottoms. For example, [26], [27] show that range size and genetic differentiation of southern Appalachian *Desmognathus* species is related to temperature-dependent resting metabolic rate, with many high elevation populations existing near their thermal maxima and significantly limited in their ability to disperse through warmer, low-elevation environments. Collectively, these studies suggest that mid and high-elevation species are generally limited to upslope migration under a warming climate, which will lead to reductions in the area occupied by those species and the extinctions of some species with small, southerly, high-elevation distributions. This is consistent with our model predictions of northward range contractions and some extinctions of southern species with small, high-elevation distributions.

We would note that even though evidence suggests that most plethodontids will be limited in their ability to disperse northward under a warming climate, our study allowed for an optimistic level of dispersal that was still not sufficient to prevent significant declines in most species. Further, we did not account for land cover and other natural or anthropogenic geographic barriers that would limit species migrations in a contemporary landscape. The southeastern United States, including the Appalachian Highlands, are predicted to have one of the largest increases in urban and exurban development in the United States with a projected population increase to more than 360 million by 2030 [53]. Large-scale reductions in climatic habitat availability combined with finer scale losses and fragmentation of remaining suitable habitats would reduce the likelihood that species could migrate with climate, increasing the probability of regional extirpations and extinction [54], [55].

The inability to account for potential evolutionary change or plasticity within the models is another potential limitation to consider. Although correlative models include variation in traits as a consequence of using occurrence data across a geographical region to model distributions, mechanistic models can be parameterized based on a representative sample of species to include variation. Identifying which traits to incorporate, data sources for model parameterization, and determining the extent of a species adaptability remains challenging [56], [57]. Although examples of species adapting to environmental change, particularly global climate change, are increasing [58], [59], little is known concerning the ability of plethodontids to adapt to changing climate conditions. Our understanding of the evolutionary adaptations or phenotypic plasticity exhibited by plethodontids to new environmental conditions is very limited. Recent studies have found correlations between genetic diversity, species diversity, and environmental variables in *Desmognathus* spp. [28], a measurable influence of moisture on adaptive phenotypes of *Desmognathus ocoee* [60], and morphological changes in *Plethodon cinereus* [61]. These studies suggest members of this family are capable of adaptation as a consequence of recent environmental change, but more conclusive evidence is needed to examine their ability to persist through adaptation.

Because of the potential pitfalls associated with species distribution modeling and forecasting, a number of studies have stressed the need to use ensemble modeling in forecasting efforts. A true ensemble approach would consider a range of algorithms to relate species distributions to climate [16], and many authors have cautioned that assumptions in species distribution models, such as the use of threshold or selection of GCM, need to be explored in forecasting efforts. Although we did not explore multiple algorithms, one strength of our study was our use of a collection of GCM, CO_2_, and threshold scenarios. While we did find that projected CO_2_ level and Maxent threshold did affect the magnitude of projected climatic habitat loss in the near term (2020), these effects were relatively small compared to the effects of current range size and latitude. One concern that has been raised regarding the use of thresholds that over-predict the current range of an organism is that the projected loss of suitable climatic habitat may be underestimated; however, our findings did not support that concern. Further, we argue that by utilizing a threshold that slightly over-predicts the current suitable climatic habitat, we allowed the climatic range of each species to be larger than realized ranges. Projected climatic habitat losses were greater for scenarios that assume high CO_2_ levels, and we note that the A2a CO_2_ emissions scenario from the IPPC 3^rd^ Assessment, which was considered a high-emissions scenario in our modeling effort, is now considered a conservative estimate of emissions [62]. In other words, our ‘high CO_2_’ scenarios using A2a emissions may be the more likely scenario for future forecasts. Remarkably, despite relatively large differences in projected temperature and precipitation changes between the Canadian and Hadley models, the choice of GCM had no measurable effect on our projected climatic habitat loss.

When using species distribution models, there are a number of additional limitations and assumptions that should be addressed [for a review see [9], [11], [14], [63]]. Biases in the availability of species distribution data, such as points concentrated within national parks or areas likely to be foci for the collection of ecological data, can bias species distribution models [41]. Our use of target-group background points has been shown to reduce sample-selection bias [41], and Maxent is considered to be a good conciliation to full ensemble forecasting [16]. Additionally, correlative, niche-based models that predict distributions solely on the association between climatic variables and species range are not explicitly mechanistic, and as discussed earlier, those models fail to account for the influence of phylogeographic or biotic processes. Mechanistic models incorporate variables of physiological requirements and limitations, and interactions of an organism's functional traits and its habitat [56], [64], [65]; however, unlike correlative models, mechanistic models require a great deal more data. For a number of taxa, data are simply not available to develop mechanistic models. Species distribution models are also affected greatly by the quality of taxonomic resolution and proper identification of species. For example, the family Plethodontidae is currently undergoing significant taxonomic revision, as detailed by the number of studies examining Plethodontidae phylogeny [52], [66], [67], [68], [69]. Revisions are particularly abundant within the genus *Desmognathus* [36], [70], [71], [72], [73], [74], [75]. Phylogenetic changes to this family in eastern North America have large implications to our study, as species currently analyzed as one single species could soon be broken into two or more species. In turn, this would separate a larger, single-species climatic niche into smaller, multiple-species niches. Based on our current models, which predicted larger percent climatic habitat loss among species with smaller geographic ranges, breaking species with larger distributions into multiple species with smaller geographic ranges and narrower climatic distributions would increase the proportion of species vulnerable to extinction and the estimated richness loss within the Appalachian Highlands.

Finally, we believe that a novel strength of our study is that our models predict measurable declines in species climatic habitat and richness as early as 2020. It is a reasonable criticism of other modeling efforts that they focus on longer-term projections (2050–2080). While longer-term projections are important for management [76], longer-range forecasts are less robust. In addition, formulating testable predictions is fundamental to science and the value of models. A number of studies have demonstrated the value of species distribution models to predicting current species distributions and patterns of richness, and then validated those models through sampling (e.g., [77], [78], [79]). To apply the same principle to species distribution model forecasting, we need shorter-term predictions of change to test. Our various 2020 model predictions can serve as testable alternative hypotheses concerning changes in species distribution and richness that will play out in the next 10 years. They also provide the opportunity to determine how other factors such as land cover change, biotic interactions, and other processes affect model projections.

The use of species distribution modeling to forecast the effects of climate change has been characterized in some ways as a necessary evil. Despite the potential pitfalls of species distribution modeling, there is a very real practical need to project how climate change may affect the distributions of species and potential losses of diversity in focal regions. We conservatively project the loss of a large proportion of plethodontid species from the southern portion of the Appalachian Highlands, a region that is currently a global biodiversity hotspot of salamander diversity. As salamanders are important in terrestrial and stream ecosystem processes [30], [31], [32], [33], the decline of species could significantly alter the function of ecosystems in that region.

## Supporting Information

Table S1 Characteristics and model results for each species modeled. Total distribution size and percent of distribution overlap of current distributions for each species with AUC values for each species to show model fit and life history traits and number of points used to model each species. Mean AUC for all species was 0.911.
(0.08 MB DOC)

Table S2 Projected change in suitable climatic habitat for each species modeled under the CGCM3 model. Percent loss or gain of suitable climatic habitat for each species using the Canadian Centre for Climate Modeling and Analysis Coupled Global Climate Model, two Maxent thresholds (strict and liberal), and two CO2 emissions scenarios (low-medium and medium-high).
(0.07 MB DOC)

Table S3 Projected change in suitable climatic habitat for each species modeled under the HADCM3 model. Percent loss or gain of suitable climatic habitat for each species using the Hadley Centre Coupled Model (version 3), two Maxent thresholds (strict and liberal), and two CO2 emissions scenarios (low-medium and medium-high).
(0.07 MB DOC)
