# Supplementary material for: Projected Loss of a Salamander Diversity Hotspot as a Consequence of Projected Global Climate Change
Source: PLoS One. 2010 Aug 16;5(8):e12189. doi: 10.1371/journal.pone.0012189 (PMC2922335; doi:10.1371/journal.pone.0012189)
Supplement: Table S1 — Characteristics and model results for each species modeled. Total distribution size and percent of distribution overlap of current distributions for each species with AUC values for each species to show model fit and life history traits and number of points used to model each species. Mean AUC for all species was 0.911. (0.08 MB DOC) [file pone.0012189.s001.doc]

**Table S1. Characteristics and model results for each species modeled.**

| Species | Life history | Size of distribution (km^2^) | Predicted current  distribution | | AUC | No. points  used to model | Centroid of species range (latitude) |
| --- | --- | --- | --- | --- | --- | --- | --- |
|  |  |  | Model threshold | |  |  |  |
|  |  |  | Strict | Liberal |  |  |  |
| *Desmognathus aeneus* | Semi-aquatic | 48219 | 28.32 | 40.40 | 0.967 | 60 | 34.03 |
| *Desmognathus carolinensis* | Semi-aquatic | 11431 | 36.19 | 56.59 | 0.982 | 57 | 35.31 |
| *Desmognathus fuscus complex* | Semi-aquatic | 1524847 | 87.00 | 95.45 | 0.764 | 923 | 39.98 |
| *Desmognathus imitator* | Semi-aquatic | 9604 | 13.29 | 44.07 | 0.960 | 283 | 35.66 |
| *Desmognathus marmoratus* | Semi-aquatic | 35121 | 58.07 | 57.62 | 0.940 | 83 | 34.76 |
| *Desmognathus monticola* | Semi-aquatic | 303109 | 61.00 | 93.33 | 0.797 | 695 | 36.43 |
| *Desmognathus ochrophaeus* | Semi-aquatic | 292260 | 82.26 | 98.95 | 0.899 | 96 | 40.31 |
| *Desmognathus ocoee* | Semi-aquatic | 42013 | 37.91 | 97.44 | 0.906 | 378 | 35.07 |
| *Desmognathus quadramaculatus* | Semi-aquatic | 84413 | 56.15 | 83.55 | 0.880 | 264 | 35.63 |
| *Desmognathus santeetlah* | Semi-aquatic | 10752 | 19.81 | 47.59 | 0.957 | 85 | 35.66 |
| *Desmognathus wrighti* | Semi-aquatic | 34505 | 7.54 | 30.75 | 0.947 | 209 | 35.95 |
| *Eurycea bislineata* | Semi-aquatic | 1198566 | 83.74 | 93.44 | 0.911 | 403 | 43.22 |
| *Eurycea cirrigera* | Semi-aquatic | 1153855 | 94.29 | 98.86 | 0.820 | 258 | 35.22 |
| *Eurycea guttolineata* | Semi-aquatic | 738309 | 94.55 | 98.14 | .927 | 57 | 33.05 |
| *Eurycea longicauda* | Semi-aquatic | 848917 | 80.08 | 99.21 | 0.892 | 126 | 34.46 |
| *Eurycea wilderae* | Semi-aquatic | 76312 | 32.21 | 73.33 | 0.842 | 668 | 35.75 |
| *Gyrinophilus porphyriticus* | Semi-aquatic | 764568 | 66.91 | 93.14 | 0.750 | 432 | 40.65 |
| *Hemidactylium scutatum* | Terrestrial | 1454021 | 62.17 | 87.24 | 0.914 | 283 | 38.75 |
| *Plethodon cinereus* | Terrestrial | 1812454 | 85.94 | 94.92 | 0.887 | 395 | 42.06 |
| *Plethodon dorsalis* | Terrestrial | 278593 | 97.70 | 93.65 | 0.994 | 45 | 35.53 |
| *Plethodon electromorphus* | Terrestrial | 113396 | 88.89 | 96.55 | 0.989 | 52 | 40.00 |
| *Plethodon glutinosus complex* | Terrestrial | 1553973 | 78.08 | 96.24 | 0.664 | 2211 | 34.53 |
| *Plethodon hoffmani* | Terrestrial | 59988 | 86.61 | 96.48 | 0.953 | 489 | 39.11 |
| *Plethodon jordani* | Terrestrial | 1943 | 49.73 | 67.47 | 0.965 | 319 | 35.61 |
| *Plethodon montanus* | Terrestrial | 7080 | 49.17 | 76.43 | 0.973 | 137 | 36.07 |
| *Plethodon punctatus* | Terrestrial | 11143 | 16.78 | 32.77 | 0.991 | 35 | 38.58 |
| *Plethodon richmondi* | Terrestrial | 100793 | 86.14 | 95.63 | 0.955 | 159 | 36.48 |
| *Plethodon serratus* | Terrestrial | 137032 | 47.06 | 93.38 | 0.907 | 291 | 37.60 |
| *Plethodon shermani* | Terrestrial | 999 | 60.04 | 61.30 | 0.983 | 81 | 35.08 |
| *Plethodon virginia* | Terrestrial | 2472 | 86.49 | 92.53 | 0.995 | 93 | 38.80 |
| *Plethodon wehrlei* | Terrestrial | 114482 | 82.02 | 92.56 | 0.953 | 189 | 38.71 |
| *Plethodon welleri* | Terrestrial | 3540 | 52.84 | 73.76 | 0.990 | 59 | 36.44 |
| *Plethodon yonhalossee* | Terrestrial | 12313 | 69.40 | 90.24 | 0.975 | 173 | 36.56 |
| *Pseudotriton montanus* | Semi-aquatic | 758984 | 81.00 | 96.66 | 0.911 | 42 | 31.70 |
| *Pseudotriton ruber* | Semi-aquatic | 1065948 | 79.57 | 96.83 | 0.747 | 570 | 36.66 |
