# Supplementary material for: Projected Loss of a Salamander Diversity Hotspot as a Consequence of Projected Global Climate Change
Source: PLoS One. 2010 Aug 16;5(8):e12189. doi: 10.1371/journal.pone.0012189 (PMC2922335; doi:10.1371/journal.pone.0012189)
Supplement: Table S3 — Projected change in suitable climatic habitat for each species modeled under the HADCM3 model. Percent loss or gain of suitable climatic habitat for each species using the Hadley Centre Coupled Model (version 3), two Maxent thresholds (strict and liberal), and two CO2 emissions scenarios (low-medium and medium-high). (0.07 MB DOC) [file pone.0012189.s003.doc]

**Table S3. Projected change in suitable climatic habitat for each species modeled under the HADCM3 model.**

|  | HADCM3 | | | | | | | | | | | |
| --- | --- | --- | --- | --- | --- | --- | --- | --- | --- | --- | --- | --- |
| Species | Strict threshold | | | | | | Liberal threshold | | | | | |
|  | B2A | | | A2A | | | B2A | | | A2A | | |
|  | 2020 | 2050 | 2080 | 2020 | 2050 | 2080 | 2020 | 2050 | 2080 | 2020 | 2050 | 2080 |
| *Desmognathus aeneus* | 0 | 100 | 100 | 95.58 | 74.36 | 100 | 0 | 100 | 99.99 | 95.17 | 80.47 | 100 |
| *Desmognathus carolinensis* | 100 | 100 | 100 | 100 | 100 | 100 | 93.99 | 100 | 100 | 100 | 100 | 100 |
| *Desmognathus fuscus* complex | 9.31 | 27.40 | 26.90 | 10.67 | 33.27 | 51.89 | 13.15 | 33.93 | 33.60 | 16.40 | 39.20 | 56.49 |
| *Desmognathus imitator* | 99.10 | 100 | 100 | 100 | 100 | 100 | 79.46 | 99.95 | 100 | 99.57 | 100 | 100 |
| *Desmognathus marmoratus* | 22.99 | 78.32 | 92.77 | 58.91 | 76.98 | 91.02 | 23.05 | 78.40 | 92.76 | 59.01 | 77.05 | 91.07 |
| *Desmognathus monticola* | 56.27 | 90.19 | 58.31 | 71.15 | 93.79 | 99.86 | 29.26 | 51.49 | 51.88 | 29.13 | 70.83 | 56.76 |
| *Desmognathus ochrophaeus* | 38.86 | 67.95 | 60.18 | 27.92 | 78.00 | 97.81 | 10.94 | 27.42 | 30.95 | 10.90 | 41.46 | 91.45 |
| *Desmognathus ocoee* | 58.01 | 95.75 | 98.92 | 86.40 | 95.04 | 97.52 | 3.48 | 89.89 | 93.28 | 51.53 | 71.88 | 93.29 |
| *Desmognathus quadramaculatus* | 73.50 | 95.47 | 95.33 | 92.12 | 97.75 | 100 | 57.79 | 92.16 | 92.18 | 85.85 | 94.48 | 99.98 |
| *Desmognathus santeetlah* | 86.14 | 99.94 | 100 | 99.86 | 100 | 100 | 71.85 | 92.03 | 98.38 | 75.36 | 94.45 | 100 |
| *Desmognathus wrighti* | 92.35 | 97.39 | 99.15 | 85.02 | 99.72 | 100 | 72.03 | 95.37 | 97.08 | 71.66 | 98.74 | 100 |
| *Eurycea bislineata* | 0 | 5.14 | 27.25 | 0 | 11.58 | 50.89 | 0 | 0 | 3.66 | 0 | 2.98 | 25.01 |
| *Eurycea cirrigera* | 22.34 | 56.19 | 55.82 | 27.86 | 60.44 | 73.87 | 23.84 | 57.30 | 57.08 | 29.41 | 61.28 | 74.61 |
| *Eurycea guttolineata* | 39.35 | 70.15 | 78.53 | 43.86 | 84.40 | 88.36 | 39.14 | 70.26 | 78.42 | 43.73 | 84.15 | 88.20 |
| *Eurycea longicauda* | 5.90 | 17.16 | 30.75 | 0 | 23.52 | 24.49 | 1.76 | 27.73 | 21.49 | 7.57 | 21.12 | 35.62 |
| *Eurycea wilderae* | 89.70 | 100 | 100 | 99.54 | 100 | 100 | 36.64 | 85.47 | 98.47 | 50.87 | 83.39 | 100 |
| *Gyrinophilus porphyriticus* | 28.57 | 63.81 | 23.42 | 21.53 | 62.61 | 80.60 | 18.13 | 20.21 | 26.19 | 11.57 | 44.75 | 37.59 |
| *Hemidactylium scutatum* | 0 | 0 | 6.88 | 0 | 0 | 15.37 | 0 | 15.14 | 16.09 | 3.93 | 20.49 | 35.76 |
| *Plethodon cinereus* | 0 | 0 | 0.00 | 0 | 8.57 | 10.06 | 0 | 2.16 | 0 | 3.25 | 10.38 | 18.46 |
| *Plethodon dorsalis* | 9.37 | 70.02 | 56.62 | 24.36 | 61.91 | 88.15 | 6.15 | 70.28 | 55.24 | 21.50 | 60.51 | 87.61 |
| *Plethodon electromorphus* | 0.00 | 20.19 | 14.40 | 0 | 0 | 0 | 0 | 44.45 | 12.76 | 0 | 0 | 0 |
| *Plethodon glutinosus* complex | 50.49 | 60.93 | 66.08 | 54.50 | 83.26 | 95.73 | 27.98 | 50.54 | 49.66 | 31.58 | 61.58 | 75.33 |
| *Plethodon hoffmani* | 93.34 | 50.94 | 95.44 | 99.24 | 100 | 100 | 57.37 | 13.78 | 55.32 | 55.32 | 96.34 | 98.78 |
| *Plethodon jordani* | 100 | 100 | 100 | 96.78 | 100 | 100 | 90.34 | 100 | 100 | 100 | 100 | 100 |
| *Plethodon montanus* | 100 | 100 | 100 | 100 | 100 | 100 | 90.70 | 99.25 | 99.98 | 99.59 | 100 | 100 |
| *Plethodon punctatus* | 100 | 100 | 100 | 100 | 100 | 100 | 99.70 | 100 | 100 | 100 | 100 | 100 |
| *Plethodon richmondi* | 66.49 | 88.30 | 64.81 | 45.88 | 88.79 | 70.64 | 43.85 | 61.31 | 43.97 | 21.96 | 46.50 | 40.73 |
| *Plethodon serratus* | 87.36 | 98.80 | 100 | 97.65 | 99.73 | 100 | 8.47 | 87.64 | 98.09 | 54.44 | 88.14 | 99.74 |
| *Plethodon shermani* | 0 | 100 | 100 | 99.05 | 100 | 100 | 0 | 100 | 100 | 96.35 | 100 | 100 |
| *Plethodon virginia* | 100 | 100 | 100 | 100 | 100 | 100 | 100 | 100 | 100 | 100 | 100 | 100 |
| *Plethodon wehrlei* | 49.38 | 80.29 | 59.49 | 66.10 | 83.23 | 99.95 | 26.98 | 60.48 | 38.65 | 41.51 | 70.66 | 99.28 |
| *Plethodon welleri* | 61.11 | 100 | 100 | 99.60 | 100 | 100 | 55.30 | 100.00 | 99.96 | 98.55 | 100.00 | 100 |
| *Plethodon yonhalossee* | 99.96 | 100 | 100 | 100 | 100 | 100 | 6.00 | 96.21 | 93.34 | 100 | 99.94 | 100 |
| *Pseudotriton montanus* | 20.49 | 59.16 | 57.57 | 22.82 | 63.42 | 77.10 | 27.68 | 63.69 | 65.05 | 29.57 | 67.03 | 80.93 |
| *Pseudotriton ruber* | 6.85 | 34.07 | 41.90 | 9.87 | 37.21 | 47.66 | 16.46 | 42.84 | 45.59 | 21.20 | 46.73 | 57.36 |
